# The Impact of Neurocysticercosis in California: A Review of Hospitalized Cases

**DOI:** 10.1371/journal.pntd.0001480

**Published:** 2012-01-24

**Authors:** Curtis Croker, Matthew Redelings, Roshan Reporter, Frank Sorvillo, Laurene Mascola, Patricia Wilkins

**Affiliations:** 1 Acute Communicable Disease Control Program, County of Los Angeles Department of Public Health, Los Angeles, California, United States of America; 2 Data Collection and Analysis Program, County of Los Angeles Department of Public Health, Los Angeles, California, United States of America; 3 Division of Parasitic Diseases & Malaria, Centers for Disease Control and Prevention, Atlanta, Georgia, United States of America; Universidad Nacional Autónoma de México, Mexico

## Abstract

To assess the burden of neurocysticercosis (NCC) in California we examined statewide hospital discharge data for 2009. There were 304 cases hospitalized with NCC identified (incidence = 0.8 per 100,000). Cases were mostly Latino (84.9%), slightly more likely to be male than female (men 57.6%, women 42.4%) with an average age of 43.5 years. A majority of cases were hospitalized in Southern California (72.1%) and many were hospitalized in Los Angeles County (44.7%). Men were more likely than women to have severe disease including hydrocephalus (29.7% vs. 18.6%, p = 0.027), resulting in longer hospitalizations (>4 days, 48.0% vs. 32.6%, p = 0.007) that were more costly (charge>$40 thousand men = 46.9% vs. woman = 4.1%, p = 0.026). Six deaths were recorded (2.0%). The total of NCC-related hospital charges exceeded $17 million; estimated hospital costs exceeded $5 million. Neurocysticercosis causes appreciable disease and exacts a considerable economic burden in California.

## Author Summary

Neurocysticercosis (NCC) is considered one of the major neglected infections of poverty in the United States, with mortality studies indicating that California bears the highest burden of this disease. Although NCC is a reportable disease in California, studies indicate that this disease goes largely under-reported, contributing to the lack of information about the disease distribution and burden. In this manuscript, we reviewed the distribution of NCC hospitalizations in California, demographics of those hospitalized and total hospital-related charges for 2009. This study revealed that a majority of persons hospitalized with NCC in California receive their medical service in Southern California hospitals, primarily in the County of Los Angeles. As compared to women hospitalized for this disease, men had a longer and more costly hospitalization with more severe symptoms such as hydrocephalus, a diagnosis suggestive of extraparenchymal infection. The reasons for this difference in NCC severity by gender are not clear, but do not appear to be due to delay in seeking medical care or a language barrier. The intensity of hospital care needed to manage these cases and the sizable NCC hospitalization charge underscores the considerable economic burden this disease presents in California.

## Introduction

Neurocysticercosis (NCC) is associated with severe disease morbidity and mortality across the globe, including the United States where its impact has been studied primarily in the states of California [1], [2], [3], [4], Texas [5] and Oregon [6]. Though NCC mortality is rare in the United States (3.9 per million population), nearly 60% of U.S. deaths occurs in California (126/221 deaths over 12 year study period) [7]. This parasitic disease is preventable, causing premature death globally and has been identified by the WHO as a potentially eradicable disease. *Taenia solium* eggs in the feces of a tapeworm carrier (taeniasis) are the source of the infection. By identifying and treating taeniasis tapeworm carriers, the risk for exposure can be eliminated. Taeniasis infection is acquired through the consumption of undercooked pork containing the larval form of *Taenia* cysts.

NCC is not reportable in most jurisdictions and data on the burden of this parasitic disease in the United States are lacking. Moreover, few population-based data sources are available. A recent published article on neglected infections of poverty has drawn attention to the need for additional data for NCC [8]. In addition, the economic impact of NCC can be sizable, with the average charge of a NCC hospitalization in Los Angeles County ($66 thousand) [1] considerably more costly than the average hospital charge in the U.S. in 2008 ($29 thousand) [9].

The clinical presentation of NCC generally takes on two forms depending on the location of the cerebral lesions [10]. Lesions appearing in the parenchymal area of the brain are characterized by seizures and associated with cerebral edema. Occasionally, these cases may develop intracranial hypertension which can be lethal without interventions such as a decompression craniotomy to allow the brain to swell. However, parenchymal NCC is generally a more benign form of NCC. In comparison, lesions forming in the extraparenchymal area of the brain often lead to a more serious form of the disease due to the obstruction and accumulation of cerebrospinal fluid in the ventricles, or cavities, of the brain causing hydrocephalus. This condition may also lead to intracranial hypertension and can be lethal without intervention such as a shunting procedure to remove excess fluid.

Several studies have pointed out that some individuals identified with NCC lesions have minimal inflammatory response to these lesions and little or no symptoms of infection, indicating that the degree of immune response and severity of illness may vary considerably by individual. An earlier study of parenchymal NCC in Mexico indicated that women with this infection present more frequently with severe illness than men [11]. Another study in Mexico also identified that females with parenchymal NCC present with more evidence of focal edema around cysticerci in CT scans as compared to men with the same infection, suggesting more severe illness among females and potentially some difference in immune response by gender to this organism [12]. In addition, this same study found little difference in the immune response by gender when reviewing extraparenchymal NCC cases, suggesting similar levels of illness severity by gender for extraparenchymal NCC.

We examined the distribution and burden of NCC in California using hospital discharge data and explored the incidence of NCC hospitalizations, demographics of those hospitalized, and factors leading to lengthy and costly hospitalizations. We also reviewed the demographics of those presenting with diagnosis suggestive of a parenchymal NCC infection as well as extraparenchymal NCC infection. Common procedures performed on NCC hospitalizations were also reviewed and the total NCC-related hospitalization charges were computed. In addition, we also describe new methodology to identify individual cases from a de-identified hospital discharge dataset that potentially contains multiple visits of the same case.

## Methods

We defined a NCC case hospitalized in California as a resident having a hospital discharge diagnosis of cysticercosis (ICD9 123.1) as a primary or additional diagnosis (1–24) AND also diagnosed with one of the following 1) hydrocephalus, seizures (including epileptic convulsions), cerebral edema or cerebral cyst. We used the 2009 California Office of Statewide Health Planning and Development (OSHPD) annual hospitalization discharge dataset for this analysis. The dataset included demographic information, primary diagnosis, up to 24 additional diagnoses and as many as 20 procedures performed while hospitalized. Also included in this dataset are length of each hospitalization and resulting hospitalization charge. This is a public use dataset and identifiers such as names have been removed.

We initially extracted data on all hospitalizations with a discharge diagnosis of cysticercosis as a primary or additional diagnosis (1–20) (ICD9 = 123.1, N = 805) from the 2009 OSHPD data set. Because this dataset contained multiple records in instances where the same case was hospitalized more than once, a sub-set was created containing one record for each case hospitalized for cysticercosis. When possible, this is done by reviewing the record linkage number (RLN; based on a coded social security number) for each visit and eliminating duplicates. Unfortunately, many of the RLN numbers for the cysticercosis hospitalization dataset created were missing (31.6%, n = 254). This may be due to the fact that cysticercosis primarily impacts an immigrant population in which many cases may not have social security numbers. To overcome this problem, an algorithm was created to assign a linkage number (RLN2) to hospital visits lacking an RLN number. This new number was constructed from demographic information available in the dataset such as age (+/−1 year), race, gender and home zip code.

This algorithm was tested against cysticercosis hospitalizations having an actual RLN number (n = 551) to assess its ability to capture duplicate records from the same case. The algorithm was successful in correctly assigning a hospitalization as either a single visit or an additional visit for 94.9% of hospitalizations; 94.7% of single hospitalizations were identified correctly and 95.3% of duplicate hospitalizations were identified correctly. We then assigned this newly constructed linkage number (RLN2) to hospitalizations previously missing a linkage number, which allowed us to create a dataset with one record for each case hospitalized, even in instances where multiple hospitalizations had been recorded (N = 670). Finally, we applied the NCC case definition described earlier to screen out any possible miscoding or incidental findings. The resulting dataset containing 304 individual NCC cases hospitalized in 2009 in CA was used for the study analysis.

The diagnostic and procedural information from cases with multiple visits was consolidated into one entry to facilitate analysis of these variables at the individual level. Incidence rates were calculated using population estimates from the California Department of Finance. SAS version 9.1 software was used for the analysis. Demographic differences in clinical illness and lengthy and costly hospitalizations were analyzed using a nonparametric chi-square testing. A lengthy hospitalization was defined as having hospitalization for more than 4 days and a costly hospital stay was defined as having a hospital charge greater than $40,000. We also analyzed the demographics of those presenting with diagnosis suggestive of a parenchymal NCC infection (seizures) as well as extraparenchymal NCC infection (hydrocephalus) using non-parametric chi-square testing. The demographics used in these analyses included gender (male, female), ethnicity (Latino, non-Latino), age (0–19, 20–39, 40–59, 60 or more years) and language spoken (Spanish, non-Spanish). Common procedures performed on NCC cases were also reviewed as well as computation of the total NCC-related hospitalization charges.

## Results

In 2009, there were 805 hospitalizations in California listing cysticercosis as a discharge diagnosis. There were 670 individual cysticercosis cases that made up these hospitalizations. Of these, there were 304 hospitalized persons that met the NCC case definition (0.8 per 100,000 persons). By comparison, 8 years earlier (2001) there were 792 total cysticercosis hospitalizations, 632 individual cysticercosis cases hospitalized and 386 persons hospitalized with NCC (incidence 1.1 per 100,000 persons).

Of the 304 NCC cases hospitalized in 2009, there were 113 cases (37.2%) discharged with a primary diagnosis of cysticercosis. Cases hospitalized for NCC were primarily Latino (84.9%) and those who primarily speak Spanish (56.9%) (**Table 1**). The prevalence of Spanish language preference was similar by gender (men = 58.3%, women = 55.0%) and by age (greater than 40 years 50.9% vs. 49.1%). Those that were age 20 to 39 years had the highest incidence of hospitalization of the age groups reviewed (1.3 per 100,000). There were 13 cases 19 years of age or younger; nine were Hispanic (69.2%) and 7 were male (53.9%). Among all NCC cases, the rate of hospitalization was slightly higher for men as compared to women (0.9 and 0.7 per 100,000 respectively). The average age of hospitalization (43.5 years) was similar by gender (men = 42.6 years, women = 44.7 years). In comparison, the average age of a NCC case hospitalized in 2001 was 38.0 years. Many of the NCC cases hospitalized in 2009 had multiple hospitalizations within the year reviewed (20.1%).

**Table 1:** Demographics of Persons Hospitalized with Neurocysticercosis in California, 2009.

Hospitalizations	n	Percent	Incidence (per 100,000)
**All**	**304**	**100%**	0.8
**Race/Ethnicity**			
Latino	258	84.9%	1.8
Caucasian	26	8.6%	0.2
African American	3	1.0%	0.1
Other or unknown	17	5.6%	-
**Gender**			
Male	175	57.6%	0.9
Female	129	42.4%	0.7
**Age Group**			
0–19	13	4.3%	0.3
20–39	134	44.1%	1.3
40–59	104	34.2%	1.0
60+	53	17.4%	0.8
**Primary Language Spoken**			
Spanish	173	56.9%	-
English	131	43.1%	-
	**Mean**		**95% CI**
Mean Age (years)	43.5		41.6–45.4
Male	42.6		40.2–45.0
Female	44.7		41.7–47.8
Median Age (years)	41.0		

Hospitalization Region*	n	Percent	Incidence (per 100,000)
Southern California	220	72.4%	0.9
Los Angeles County	136	44.7%	1.3
Orange County	26	8.6%	0.8
San Diego	24	7.9%	0.8
San Bernardino	13	4.3%	0.6
Kern	12	3.9%	1.5
Northern California	84	27.6%	0.6
Santa Clara	14	4.6%	0.8
Alameda	8	2.6%	0.5

*Northern CA; 48 northern most counties. Southern CA; 10 southern most counties.

Population data obtained from the CA Department of Finance.

The majority of NCC cases in California were hospitalized in the Southern California region (72.4%, 0.9 per 100,000) which included the counties of Los Angeles (44.7%, 1.3 per 100,000), Orange (8.5%, 0.8 per 100,000) and San Diego (7.9%, 0.8 per 100,000). Many of the NCC cases hospitalized in Northern California occurred in the counties of Santa Clara (4.6%, 0.8 per 100,000) and Alameda (2.6%, 0.5 per 100,000).

Additional diagnoses identified among NCC cases included seizures (74.3%), hydrocephalus (25.0%), cerebral cyst (7.6%) or cerebral edema (6.6%). Some cases were diagnosed with both hydrocephalus and seizures (7.5%). Many patients received a neurological procedure while hospitalized (41.0%) which included cranial procedures (25.0%) such as a ventricular shunt procedure (14.8%) or the excision [or destruction of] a brain lesion (8.2%). Some of these shunt procedures involved the removal and replacement of a ventricular shunt (6.3%). The average length of a hospital stay was 6.5 days and the average hospital charge was $57.8 thousand dollars. Six deaths occurred during the period of hospitalization (2.0%). Deaths occurred among both men (n = 3) and women (n = 3) and all were Latino. Ages of deaths ranged from 33–71 years (median 50.5 years). One death had hydrocephalus and had received a shunt procedure. One death had cerebral edema and 4 others had diagnosis of seizure. Other health conditions frequently listed among NCC cases include diabetes (15.5%) and heart disease (12.8%). One case was also diagnosed with taeniasis.

**Figure 1:**
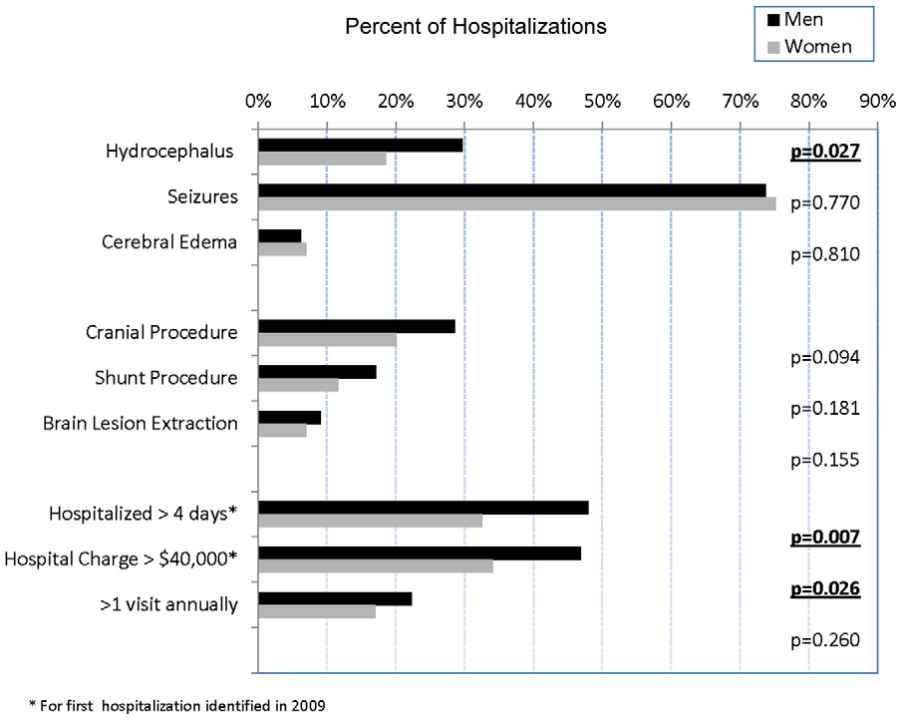
Neurocysticercosis hospitalizations; additional diagnoses, procedures performed and hospital utilization by gender, CA 2009 (N = 304). This figure shows that men hospitalized with neurocysticercosis have more severe symptoms requiring a longer and more costly hospitalization than women with neurocysticercosis. Specifically, men hospitalized with neurocysticercosis were more likely to have an additional diagnosis of hydrocephalus, to have a hospitalization exceeding four days and to have a hospital charge exceeding 40,000 dollars as compared to women hospitalized with neurocysticercosis.

Overall, 41.5% of NCC cases had lengthy hospital stays (>4 days). Men were more likely to have a lengthy stay as compared to women (men = 48.0% vs. woman = 32.6, p = 0.007, **Figure 1**), but age and race ethnicity were not associated with lengthy stays, nor was language spoken. NCC cases with hydrocephalus were more likely to have a lengthy stay as compared to cases without hydrocephalus (63.2% vs. 34.2%, p<0.001), but cases with seizures were not associated with a lengthy stay (37.2% vs. 53.9%), nor were those with cerebral edema (35.0% vs. 41.9%). Chronic health conditions such as heart disease were associated with a lengthy hospital stay (59.0% vs. 38.9%, p = 0.017). Of interest, heart disease was no more likely to be diagnosed among men than women with NCC (men = 10.9%, women = 15.5%). Other chronic health conditions such as diabetes were not associated with a lengthy hospital stay (46.8% vs. 40.5%). Multiple hospitalizations, another measure of health care utilization, was not associated with gender (men = 22.3%, woman = 17.2%, **Figure 1**) or age, race ethnicity or language spoken.

The percent of NCC cases having a costly hospitalization (charges >$40,000) was 41.4%. By gender, men were also more likely than woman to have had a costly hospitalization (46.9% vs. 34.1%, p = 0.026, **Figure 1**), but other demographics such as age, ethnicity and language spoken were not associated with a costly hospitalization. NCC cases with hydrocephalus were associated with a costly hospitalization as compared to those without hydrocephalus (68.4% vs. 32.5%, p <0.001), however cases with seizures were not associated with a costly hospitalization (32.3% vs. 68.0%), nor were those with cerebral edema (40.1 vs. 60.0%). NCC cases with heart disease were also associated with a costly hospitalization (59.0% vs. 38.9%, p = 0.002), however those with diabetes were not associated with a costly hospitalization (46.8% vs. 40.5%).

NCC cases with hydrocephalus were associated with male gender (men = 29.7%, women = 18.6%, p = 0.027, **Figure 1**) and associated with having an age greater than 20 years [no cases under the age of 20 years identified]. Aside from this finding, hydrocephalus was not associated with ethnicity, language spoken or other age groups. Among those diagnosed with hydrocephalus (n = 76), men were more likely to be associated with a lengthy hospitalization (>4 days) than women (men = 71.2% vs. women = 45.8%, p = 0.03). However, there was no association with costly hospitalizations (>$40,000) among NCC cases with hydrocephalus by gender (men = 71.2%, women = 62.5%). Age, race ethnicity and language spoken were not associated with lengthy stays among NCC cases with hydrocephalus.

NCC cases with seizures were not associated by gender, age, ethnicity or language spoken. By gender specifically, seizures appeared at very similar rates (men = 73.7%, women = 75.2%, **Figure 1**). Among NCC cases with seizures (n = 226), men appeared to have a more lengthy hospital stay (44.2% vs. 27.8%, p = 0.011), but this finding was not significant when removing NCC cases who also had hydrocephalus from the analysis (n = 209, 39.1% vs. 28.7%). Among NCC cases with seizures (n = 226), a costly hospitalization was not associated with gender (men = 36.4% vs. women = 26.8%) or by age, race ethnicity or language spoken. NCC cases with cerebral edema (n = 20), also a diagnosis suggestive of a parenchymal infection, was not associated by gender (men = 55.0% vs. women = 45.0%, **Figure 1**) or age, ethnicity or language spoken. NCC cases with cerebral edema were not associated with more lengthy hospital stays (35.0% vs. 41.9%) or more costly hospital stays (60.0% vs. 40.1%).

Cranial procedures performed on hospitalized NCC cases was not associated with gender (men = 28.6% vs. women = 20.2%, **Figure 1**), age, ethnicity or language spoken. Shunting procedures were not associated by gender (men = 17.1% vs. women = 11.6%) age, ethnicity or language spoken. No shunting procedure was performed on NCC cases less than 20 years of age. Shunting procedures were almost exclusively performed on cases with hydrocephalus (97.8%). A shunting procedure was associated with having multiple hospitalizations (29.5% vs. 11.1%, p<0.001). NCC cases having a brain lesion extraction were not associated by gender (men = 8.6%, women = 7.8%) age, ethnicity or language spoken. NCC cases requiring a brain lesion extraction (n = 25) involved cases with hydrocephalus (44.0%), seizures (52.0%) and cerebral edema (8.0%), conditions not mutually exclusive.

In summary, the demographic that appeared the most consistently associated with illness severity in this analysis was male gender. A summary of severity indicators such as diagnosis, procedures and hospital utilization are shown in Figure 1.

## Discussion

Our findings support the notion that NCC remains a significant problem in California, especially in Los Angeles County (LAC), and the burden of severe disease and economic costs are considerable. The number of hospitalized cases reported here (n = 304) far exceeds the number reported to the California State Health Department (n = 32) for 2009 [13] and illustrates the considerable under-reporting of NCC. The incidence of NCC hospitalization in 2008 in California is comparable to that found in 2001, indicating that the burden of disease has not changed much in the last 8 years in California. The largest majority of cases were among Latinos from Southern California counties. However, NCC hospitalizations were observed across racial ethnic groups and in 29 California counties.

The proportion of hospitalized NCC cases that were identified as Latino in this study (84.9%) was slightly lower than that identified in another study of NCC mortality in California (92.7%) [2]. The average age of a NCC case hospitalized in California for 2009 (43.5 years) was older than that seen among hospitalized NCC cases in 2001 (38.0 years) and also much older than that found in earlier studies of hospitalized cases in LAC. One study of hospitalized NCC cases in four hospitals in LAC (1973–83) identified an average age of 31.1 years [3]. A study of cysticercosis deaths in California (1989–2000) identified a mean age of 34.5 years [2]. The number of repeat hospitalizations per year identified in this study is also larger than that found in another more detailed study of hospitalized NCC cases in a single Texas hospital (20% vs. 14%) [5]. These findings underscore the chronic and debilitating nature of this disease. The results of our study may indicate a shift in the demographics of the disease from a younger age, more acute illness, to older age, more chronic illness. It may also indicate that the incidence of NCC in endemic regions where cases are migrating from may be declining [14].

Our study found that the severity of NCC in California was associated with male gender. Men were slightly more likely to be hospitalized for NCC (58%). Other studies of reported cases have also shown similar results; 54% male (LAC 1973–83) [3] and 58% male (LAC 1981–88) [7]. Our study revealed that men with NCC have longer and more costly hospitalizations as compared to women hospitalized with the same infection. In addition, men were found to have more severe disease that includes hydrocephalus, suggestive of a higher incidence of extraparenchymal infection among men. These findings are consistent with the finding of another study of NCC mortality in California indicates that men have a mortality rate nearly twice that of women in CA (5.2/10^6^ vs. 2.7/10^6^) [2]. This study also found that 31% of deaths occur outside of a hospital setting and would not be captured in our analysis. However, our findings differ from an earlier study involving a Mexican study population suggesting that the inflammatory response to extraparenchymal NCC infection does not differs by gender, implying that severity of extraparenchymal NCC should not differ by gender [12]. This earlier study also suggests that the inflammatory response to parenchymal NCC is more severe among women, where our study found little evidence to support differences in severity of parenchymal infection by gender. Some of these discrepancies may be the result of very different study populations. The study group in Mexico [12] was more likely comprised of NCC cases with locally acquired infection whereas our study population is more likely to be comprised of immigrants with exposure outside of the U.S. The more urgent economic necessity for men to migrate to California for employment may explain some of the difference in the study findings.

This difference in NCC severity by gender does not appear to be the result of gender differences in other chronic health conditions such as heart disease or diabetes that could results in more lengthy and costly hospitalizations. Could it be that men delay seeking medical attention until their condition is more severe? However, if this were true, we might expect the average age of men hospitalized for NCC to be older than women, which was not the case. Is it due to a language barrier in understanding the management of this complicated disease? Is it that men receive less care, or opt for less care once hospitalized? Our findings suggest that this is also not the case. It may also be possible that the difference in disease severity by gender identified in our study is due to a difference in immune response to NCC as suggested in this earlier study [12].

This is the first study that documents a method of managing a de-identified public use hospital discharge dataset that is missing a large number of RLN's. This is especially important for analyzing severe chronic diseases such as NCC where persons may have multiple visits within the same year. As was identified in an earlier study [1], the economic impact of NCC is sizeable. We found that the total charges associated with NCC hospitalizations in California exceeding $17.1 million annually. The average charge for a NCC hospitalization in California for 2009 ($57.8 thousand) was significantly higher than that of the average hospitalization charge in the U.S. ($30.6 thousand) [9]. Based on the average charge to cost to ratio for a hospitalization nationally in 2009 (3.0∶1, n = 39,434,956) [9], the estimated total cost of NCC hospitalizations in California would be $5.1 million. The estimated average cost of a NCC hospitalization in California in 2009 would be $17.3 thousand. The average length of a NCC hospitalization in 2009 (6.5 days) was also considerably longer than that of the average hospitalization in the U.S. (4.6 days) [12]. The additional costs, beyond hospitalization, of emergency room visits, outpatient care, disability and days of work lost all contribute to the overall financial impact of NCC.

Any conclusions based on hospital discharge data may be limited for several reasons. It is possible that persons without an RLN (SSI) number may be more likely to relocate and have a change in zip code within a given year. This may cause multiple hospitalizations by the same cases to be interpreted as separate persons hospitalized when using the described method. Also, cases listed here as having only one hospitalization may have other hospitalizations in the previous year that would not be captured due to the selected study period. In addition, hospitalizations of California residents outside of California would not be captured by our data.

The hospital charges presented here may overestimate the actual cost of hospitalization, as the actual amount received for hospital services may be less than the amount charged. However, we feel that the calculation of hospital charges can be useful when used in comparison to an average hospital charge in the U.S. and to hospital charges for other specific diseases.

It would be helpful to know how much transmission is occurring in the US; however, this dataset does not have any information on the case's country of origin and how long cases have resided in the US. Thus there is no way to know if any of these infections were acquired locally verses their country of origin or through possible exposure when traveling.

There is no information in the dataset regarding the type of laboratory diagnostics used to make these diagnoses, making it difficult to know how confident we can be in these diagnoses. It is also possible that a patient may have been clinically diagnosed with cysticercosis, but were not identified as such in the ICD9 discharge codes.

Finally, while we identified the number of incident hospitalizations for cysticercosis in this study, a distinction should be made between incident hospitalizations and incident cases. This dataset does not allow us to determine whether the hospitalization represents a newly diagnosed case or a previously identified case.

In Conclusion, neurocysticercosis exacts a significant disease and economic toll in California, especially in Los Angeles County. Hospitalized NCC cases are primarily working age Latinos. As compared to women, men are hospitalized with more severe disease and more costly and longer hospital stays. The reason for this difference in severity by gender is unclear, but potentially gender should be considered by physicians for proper disease management. NCC is a preventable disease and public health efforts to identify and treat the tapeworm carrier could be improved and adopted by other public health systems. Increased reporting from hospitals and clinicians in California will allow for public health follow-up of cases, identification and treatment of *T. solium* tapeworm carriers, prevention of additional cases, and reduction of the disease burden in California.

## Supporting Information

Checklist S1 Strobe checklist.
(PDF)
